# Assessment of Volumetric versus Manual Measurement in Disseminated Testicular Cancer; No Difference in Assessment between Non-Radiologists and Genitourinary Radiologist

**DOI:** 10.1371/journal.pone.0168977

**Published:** 2017-01-12

**Authors:** Çiğdem Öztürk, Ton Velleman, Alphons H. H. Bongaerts, L. M. Bergman, Robert J. van Ginkel, Jourik A. Gietema, Harald J. Hoekstra

**Affiliations:** 1 Department of Surgical Oncology, University of Groningen, University Medical Center Groningen, Groningen, the Netherlands; 2 Radiology, University of Groningen, University Medical Center Groningen, Groningen, the Netherlands; 3 Medical Oncology, University of Groningen, University Medical Center Groningen, Groningen, the Netherlands; Northwestern University Feinberg School of Medicine, UNITED STATES

## Abstract

**Background:**

The aim of this study was to assess the feasibility and reproducibility of semi-automatic volumetric measurement of retroperitoneal lymph node metastases in testicular cancer (TC) patients treated with chemotherapy versus the standardized manual measurements based on RECIST criteria.

**Methods:**

21 TC patients with retroperitoneal lymph node metastases of testicular cancer were studied with a CT scan of chest and abdomen before and after cisplatin based chemotherapy. Three readers, a surgical resident, a radiological technician and a radiologist, assessed tumor response independently using computerized volumetric analysis with Vitrea software® and manual measurement according to RECIST criteria (version 1.1). Intra- and inter-rater variability were evaluated with intra class correlations and Bland-Altman analysis.

**Results:**

Assessment of intra observer and inter observer variance proved non-significant in both measurement modalities. In particularly all intraclass correlation (ICC) values for the volumetric analysis were > .99 per observer and between observers. There was minimal bias in agreement for manual as well as volumetric analysis.

**Conclusion:**

In this study volumetric measurement using Vitrea software® appears to be a reliable, reproducible method to measure initial tumor volume of retroperitoneal lymph node metastases of testicular cancer after chemotherapy. Both measurement methods can be performed by experienced non-radiologists as well.

## Introduction

Diagnostic process for testicular cancer (TC) includes physical examination, laboratory tests for tumor markers alpha-fetoprotein (AFP), human chorionic gonadotropin (HCG), lactate dehydrogenase (LDH), and imaging tests, such as testicular ultrasound and computed tomography (CT) of abdomen and chest to assess regional and distant metastases. CT scans and tumor marker analysis make it possible to stage the extensiveness of disease and to classify the patient according to the International Germ Cell Consensus Classification (IGCCC) [1,2]. Staging results are fundamental in determining the prognosis and the optimal treatment strategy for each individual patient. Stage I non-seminomatous testicular germ cell tumor (NSTGCT) can be treated successfully with a wait-and-see policy, or unilateral nerve-sparing retroperitoneal lymph node dissection (RPLND), or one course of adjuvant cisplatin based chemotherapy after orchidectomy [2]. Disseminated disease is treated also with three or four courses of cisplatin-based chemotherapy. Resection of residual retroperitoneal tumor masses (RRRTM) after chemotherapy is an essential part of the combined therapy of NSTGCT [3,4]. CT of chest and abdomen serve as the main diagnostic tool to determine pre- and post-chemotherapy tumor deposit size, allowing evaluation of the response to treatment and guiding the decision to perform RRRTM, either conventional or via laparoscopic surgery [5]. An alternative approach to surgery can be observing patients after systemic chemotherapy, certainly if abdominal residual tumor masses have become undetectable (lesions < 1 cm) on CT scans and in some institution if the primary tumor did not contain teratoma components [6,7]. Residual metastatic disease after completion of chemotherapy can contain tumor necrosis, teratoma or viable tumor.

Optimal disease management is based on reliable and reproducible lesion measurement. Tumor response assessment has been standardized since the introduction of the bi-dimensional response criteria of the World Health Organization (WHO) which was more recently followed by one-dimensional (2D) response criteria based on RECIST (version 1.1), which is a set of rules defining overall tumor burden at baseline and objective tumor response and disease progression after systemic treatment [8,9].

In short, 2D measurements are obtained to assess the size of a tumor lesion. Measurements are obtained in the axial plane on a single slice, after careful selection from all axial planes in which the tumor lesion is visible. Assuming such single slice manual measurements in the axial plane comprise complete change in tumor size, neglects the fact that a tumor is an irregular three-dimensional lesion which is not a perfect sphere and therefore can be a misinterpretation of tumor burden. Additionally, in literature high inter- observer and intra-observer variability in manual tumor assessment are reported because of the difficulty to define the outer borders of the lesion to be measured and the variation in perception of the largest diameter [10,11].

An approach to improve reproducibility of lesion size measurements, and to reduce time and effort of measurements, combined with rapid technological developments, have led to the development of software allowing semi-automated lesion segmentation of tumor masses [12,13]. Semi-automatic segmentation tools are utilized to acquire 3 dimensional (3D) images and accurate determination of tumor size alterations. Opposed to manual 2D measurements, in this 3D setting, asymmetrical tumor size changes can be detected and measured. Lymph node metastases are irregularly shaped and often closely located to adjacent tissue with similar contrast density. These characteristics, in combination with anatomical location and slice thickness, influence segmentation quality and increase measurement error, especially when measuring smaller shaped lesions [14,15]. The spread of TC to lymph nodes usually follows a predictable pattern with metastases occurring in retroperitoneal lymph nodes. Lymph node metastases in this region can be quite difficult to measure even with adequate contrast administrations and regular manual measurements, because of surrounding tissues with similar contrast densities causing indistinct borders of the tumor deposits [16]. Volumetric analysis is a new promising technique to measure a therapeutic response especially for lung and liver lesions, which can also be used to measure response of retroperitoneal lymph nodes of testicular cancer after chemotherapy [17].

The purpose of this pilot study was to assess the suitability and reproducibility of semi-automated volumetric analysis when applied to retroperitoneal lymph nodes in patients with disseminated TC, compared to 2D measurements based on RECIST between non-radiologists and a genitourinary radiologist.

## Materials and Methods

We consulted the Institutional Review Board (IRB) of the UMCG, and they confirmed that no formal written waiver for the need of ethics approval was required because of the retrospective design of the study. Also there was no written consent needed from the patients.

One of the supervising authors has had contact with some of the study participants as the surgeon who performed the resection of the lymph node metastases and therefore was a treating physician during a short period. This author did not perform the manual and volumetric measurements.

Authors did not collect any personally-identifying information from study participants. Furthermore, all data were detached of patient information prior to analysis.

### Study population

CT scans of Chest and Abdomen of 21 consecutive non-seminomatous testicular cancer patients (NSTGCT), treated at the University Medical Centre Groningen (UMCG), with a mean age of 36 (range 27–68) years, undergoing resection of residual retroperitoneal tumor mass (RRRTM) after cisplatin based combination chemotherapy were evaluated. Of the 21 patients, 16 patients (76%) had stage II disease, 2 patients (10%) had stage III disease and 3 patients (14%) had stage IV disease. According to the International Germ Cell Consensus (IGCC) classification the majority of patients, 15 (71%) had disease with good prognosis, 4 patients (19%) intermediate prognosis and 2 (10%) poor prognosis.

Lesions outside the retroperitoneal area were not considered for this analysis. All patients had pre-and post-chemotherapy CT imaging performed at the UMCG or the referring hospital.

Finally 21 patients were eligible with 28 individual lesions identified in the retroperitoneum.

### Data collection

All post-chemotherapy CT scans were performed at the UMCG with a multi-slice CT scanner (Sensation S64, Siemens Healthcare, Forchheim, Germany) according to the TC protocol, which encompasses fasting 4–6 hours prior to the scan after which scanning of abdomen and thorax is performed with oral and intravenous contrast (100cc/flow 2.5). The reconstructed slice thickness for the standard protocol was 2.0 mm [18,19].

The majority of the pre-chemotherapy CT scans were performed in the referring hospitals. Depending on CT scanner quality and primary indication to perform a CT scan, reconstructed slice thickness was 2 mm for all scans.

All data were transferred to a dedicated advanced visualization workstation and were analyzed with a software package of Toshiba systems (Vitrea®/Vital Images, version 4) [20]. Vitrea® software is Vital Images' visualization software and commercially available for implementation. With this software 2D, 3D and 4D images are created of human anatomy from CT, MR (magnetic resonance) and PET (positron emission tomography) image data. The software provides options for cardiac, colon, vessel probe and other applications.

### Evaluation of retroperitoneal lymph node metastases

All manual and semi-automatic measurements of the labeled retroperitoneal lymph node metastases were executed independently by 2 non-radiologists (surgical resident and radiological technician) blinded for patient data and each others readings. These measurements were performed on a separate workstation without access to patient records. After transfer of CT scans to this separate workstation (without attachment of patient records), the readers measured the defined lesions while evaluating the CT images and performed the measurements. Tumor size was assessed based on RECIST criteria (1.1) [8,9].

**Fig 1:**
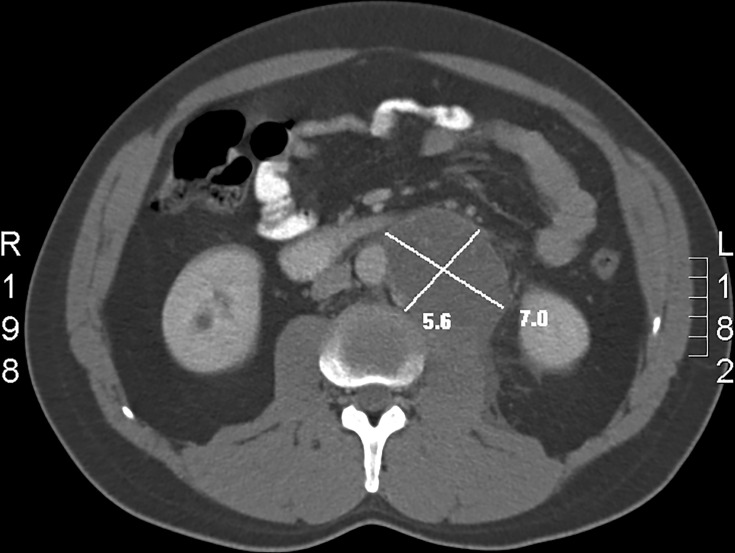
Measurement according to RECIST in one dimension. Long axis and short axis measurement in cm is shown in the figure.

The axial plane of the images chosen individually by each reader was used to draw long axis (LAD) and short axis (SAD) diameters in millimeters (Fig 1).

**Fig 2:**
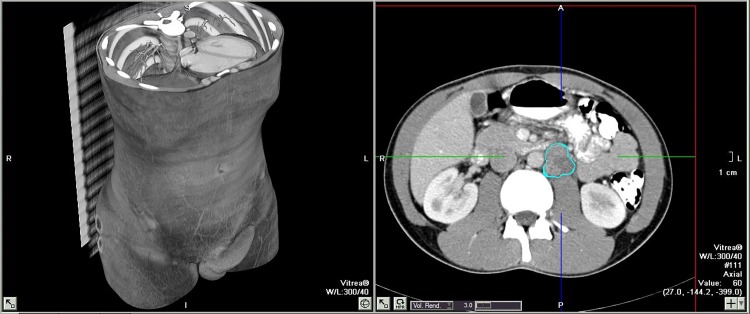
Vitrea® semi-automatic volumetric analysis. Contour of the retroperitoneal lesion is marked.

Semi-automatic evaluation of the retroperitoneal lymph nodes was also performed independently by the readers with Vitrea® software. This software package provided tools to perform volumetric measurements by semi-automatic segmentation on retroperitoneal lesions by manually selecting lesions in the axial and coronal plane followed by linear interpolation to create a 3D view. A nodule segmentation algorithm, based on radiodensity and grey scale differentiation, using Hounsfield unit values, is applied to separate the target lesions from background tissues and also morphological procedures are applied to separate these target lesions from adjacent tissue of the retroperitoneum. A visual assessment then followed on multi-planner reconstructions determining the contour of the lesion and in case of unsatisfactory segmentation manual adjustments could be made with correction tools if the lesion was not fully included in the segmentation field or if there was too much overlap of adjacent tissue (Fig 2). After satisfactory segmentation results, the software automatically generated and displayed a volume in cubic centimeters (cc).

Finally, an experienced genitourinary radiologist also performed manual 2D and semi-automatic 3D measurements to make comparisons versus measurements made by the non-radiologists.

### Statistical analysis

Statistical analysis was performed with SPSS software version 18, City, US, Microsoft Excel, City, US.

Measurement meant either the tumor diameter in mm or volume in cc. In order to attain geometrically equivalent entities, the sphere volume formula V = π D^3^ /6 was used to convert all volume measurements from mm^3^ to effective (spherical) diameters in mm.

Mean changes and range were calculated with SPSS. Descriptive statistics were used to summarize lesion characteristics and parameters (LAD, SAD, volume).

Intraclass correlation (ICC) values were used to assess intra-observer variation in the measurements according to RECIST and the volumetric method. Correlations were calculated to examine associations between the two measurement methods. Correlations with a coefficient <0.30 were considered weak, between 0.30–0.50 moderately strong, and >0.50, strong [21].

Intraclass correlation values were also applied to assess inter-rater variability of the two measuring methods. Furthermore, Bland-Altman analysis was performed to determine and visualize the inter-rater agreement between 2 readers. Bias (average difference between 2 readers), 95% confidence intervals and 95% limits of agreement were calculated.

## Results

### Radiologic characteristics of the retroperitoneal lymph nodes

**Fig 3:**
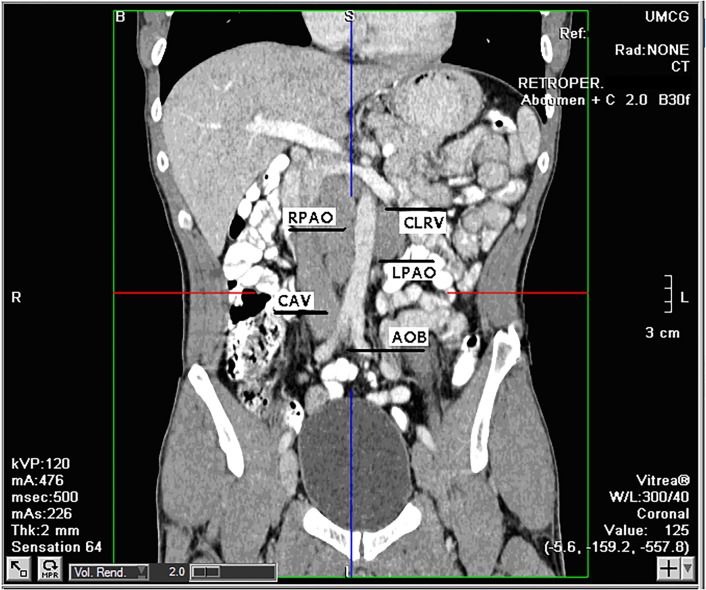
Distribution of retroperitoneal lymph nodes. RPAO: right para-aortic, LPAO: left para-aortic, CLRV: caudal left renal vein, CAV: vena cava, AOB: aortic bifurcation.

Evaluation of CT scans of 21 patients, all diagnosed with NSTGCT, led to the identification of 28 individual retroperitoneal lymph nodes, which were analyzed manually and semi-automatically. Mean number of lymph nodes per patient was 1.5 (range 1–3). Distribution of lymph node size is presented in Table 1 and Fig 3. All these patients were in biochemical complete remission with normal tumor marker levels at time of the restaging CT-scan. Retroperitoneal lymph nodes which were resected either through a laparoscopic procedure or through a conventional laparotomy were localized as follows: 10 (35%) left para-aortic, 1 (4%) right para-aortic, 2 (7%) aortic bifurcation, 14 (50%) caudal of the left renal vein and 1 (4%) vena cava.

**Table 1:** Patient characteristics and lesion distribution.

Patient characteristics	
Patients (n)	21
Age median (range)	31(22–63)
Lesions (n)	28
Stage II	16(76%)
Stage III	2(10%)
Stage IV	3(14%)
IGCC good	15(71%)
IGCC intermediate	4(19%
IGCC poor	2(10%)
Anatomical site	
RPAO (n)	1
CLRV (n)	14
LPAO (n)	10
CAV (n)	1
AOB (n)	2

Table 2 provides an overview of the distribution of manual and volumetric measurement results as measured by 3 readers.

**Table 2:** Distribution of manual and volumetric lymph node measurements (mm).

Parameters	Observer 1	Observer 2	Radiologist
**Pre chemo**			
LAD Mean ± SD	27.6±14.2	28.1±17.5	27.2±13.5
LAD Median (range)	26.5 (8.2–76.4)	24.8 (7–91.3)	26.5 (5–70)
SAD Mean ± SD	24.3±14.8	23.5±13.5	23±12
SAD Median (range)	21.8 (4–75.9)	20.7 (6–67)	21.5 (5–56)
Volume Mean ± SD	26.2±13.1	25.7±12.7	24.7±12.9
Volume Median (range)	24.1 (8–70)	23.5 (8–68)	22 (7.8–69.8)
**Post chemo**			
LAD Mean ± SD	21.9±14.4	21.3±13.7	21.1±14.3
LAD Median (range)	17.9 (7.5–64.1)	16.7 (7–60)	17 (6–60)
SAD Mean ± SD	18.9±13.5	17.9±13.6	17.8±13.7
SAD Median (range)	14.6 (4.5–56.3)	13.5 (5.3–59)	13.5 (5–60)
Volume Mean ± SD	20.7±11.9	20.5±11.6	19.2±11.7
Volume Median (range)	17.3 (6.9–51.7)	16.9 (7.1–51.4)	15.5 (6.7–50.4)

### Intra-observer variability: reproducibility by the same reader

Retroperitoneal lymph nodes were measured three times by 2 non-radiologist readers on the defined lesions. The ICC correlation for both readers was very significant with a correlation coefficient of .99 for reader 1 versus .98 for reader 2 with a corresponding P-value of < .001 for both readers.

Volumetric analysis was repeated three times for each individual lymph node by the two readers showing an almost 100% concordance for the individual measurements in axial and coronal plane. All ICC values were >.99 with a p-value of < .001.

### Inter-observer variability: consensus between readers

#### Absolute measurements: manual and volumetric method compared

**Fig 4:**
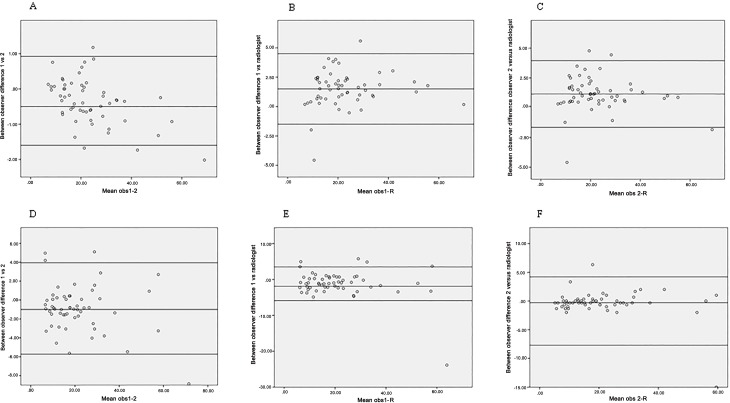
Bland Altman plots. **(A)** Display Bland Altman plot observer 1 and 2 volumetric method (cc). (B) Display Bland Altman plot observer 1 and R (radiologist) volumetric method (cc). (C) Display Bland Altman plot observer 2 and R volumetric method (cc). (D) Display Bland Altman plot observer 1 and 2 manual method (mm). (E) Display Bland Altman plot observer 1 and R manual method (mm). (F) Display Bland Altman plot observer 2 and R manual method (mm). Mean difference and limits of agreement are displayed as reference lines.

Mean of the absolute measurements was used to calculate ICC values between two readers (Table 3). ICC values show an excellent correlation for both measurement methods between these readers with a p-value of <0.001. Bland-Altman analysis (Fig 4) for testing the degree of agreement for manual as well as volumetric analysis shows minimal bias. Measurements of both methods of the two readers were compared with measurements performed by an experienced radiologist. In Table 3 high ICC values are displayed. Results of the Bland Altman analysis are shown in Fig 4A–4F. In this graphical representation degree of agreement is displayed with 95% limits between observers on both manual and volumetric measurements. From these plots it was concluded that the spread between points are between the limits of agreement for both methods.

**Table 3:** Overview intra class correlation values between observers across methods.

Volumetric	ICC	P-value
Observer 1–2	.99	.000
Observer 1-radiologist	.99	.000
Observer 2- radiologist	.99	.000
**Manual**		
Observer 1–2	.97	.000
Observer 1-radiologist	.96	.000
Observer 2-radiologist	.98	.000

#### Required time to perform volumetric analysis

Time needed to perform volumetric measurements was calculated for 2 readers. Median measurement time for reader 1 was 1.42 (0.37–3.66) minutes and for reader 2 was 2.69 (range 1.19–4.54) minutes. Time of lymph node selection and transfer to another workstation where Vitrea® analysis could be performed was approximately 10–15 minutes. Time to perform RECIST was not measured. Characteristics of the measurements of the radiologist are lacking.

## Discussion

Measuring tumor burden and tumor response to therapy as accurate as possible in oncological practice remains a challenge. Limitations of manual measurements according to RECIST have been described in literature. An important limitation is that tumor measurement occurs at differently chosen slides by the readers, leading to a large intra- and inter-observer variation [10,11]. Volumetric images have gained importance in medical applications in recent years, resulting in software products allowing volumetric analysis. In liver surgery volumetry is important to calculate postoperative liver volume and liver function resulting in safety enhancement of liver resection procedures [12,22,23].

In the present study, radiological tumor response after chemotherapy in patients with disseminated non-seminomatous TC with retroperitoneal lymph node metastases was evaluated using two measurement procedures, namely 2D measurements based on the RECIST criteria and semi-automatic volumetric analysis with specialized computer software.

Results show a high intra-observer agreement for the measured tumor volumes with the used software package. Measurement of the retroperitoneal lymph node volumes performed by each reader, both non-radiologists, showed also high correlations. This corresponds with findings of semi-automatic volumetric analysis in lung nodules which are promising and show reproducible measurements with high accuracy [24–26]. In the present study intra-observer agreement for the 2D manual measurements was also high with ICC values of .99. In a previous study concerning intra-observer variability in the response evaluation according to RECIST in solid tumors such as breast cancer and colorectal cancer, correlation values ranged between 0.76–096 [27]. Intra-observer agreement with these manual measurements in the current study was higher than expected based on previous literature on this subject.

In the present study, user experience was assessed by measuring inter-observer agreement. Measurements were performed by non-radiologists as well as a radiologist to evaluate its user friendliness and reliable application in the routine hospital. Inter-observer variability for semi-automatic measurements was comparable for manual measurements, with acceptable limits of agreement. When we looked at absolute measurement values both measurement modalities were highly reliable. In another study, inter-observer variability was significantly lower in the semi-automatic method [28].

Good results were obtained with volumetric analysis with a high level of agreement between observers and methods. It should be taken into account that the complexity of the parameters still require a lot of work to produce an informative and reliable image. Also, volumetric analysis of lymph node metastases compared to, for example, lung nodules can be more difficult since lymph node metastases are often irregularly shaped with inhomogeneous contrast enhancement and are in the proximity of similar density tissues.

Tumor volumetry can be time consuming because of the segmentation process. However in experienced hands of, a radiologist, a radiological technician or a resident the time investment seems appropriate. Also, results from this study showed that novices with some radiological experience and an experienced assessor such as a radiologist were both able to reliably predict tumor volume using the semi-automatic computer software. This validation study has been conducted in a center of expertise for patients with testicular cancer for over more than three decades. In present times there is a paradigm shift in the radiologic evaluation of lesions. Where in the past the executing surgeon relied 100% on the readings of a radiologist, nowadays the surgeon and a surgical team need tools to get a quick and reliable impression of lesions before performing a specific surgery. We have found that the volumetric method can be performed by an experienced non-radiologist as well and that the required time to obtain and measure a reliable 3D image is under 3 minutes. Such a study for volumetric measurements is lacking in literature for retroperitoneal lymph node metastases in testicular cancer. The new technology facilitates the surgical-oncologist or the uro-oncologist in the conventional or laparoscopic surgical treatment planning of the resection of tumor masses.

In conclusion, the pilot study showed that radiological technicians and (surgical/urology) residents could perform volumetric and manual measurements in a reliable manner with a high intra and inter-observer reliability. With the increase in the number of cancer patients receiving chemotherapy, automatic and semiautomatic segmentation tools will likely play an important role in the evaluation of tumor response to treatment, e.g. a cost-effective way to measure tumor response and prepare the surgical or uro-oncologist in the resection of retroperitoneal lymph node metastases. For now, clinical guidelines based on manual measurement in 2D in the axial plane according to RECIST criteria remain the standard method for assessing a radiological tumor response to anti-cancer therapy. This study has shown that the volumetric method is reproducible, user friendly and is promising as a more accurate method in the surgical field since it provides a 3D view. Especially the latter can make a clinical difference with more detailed surgical planning and can contribute to achieving better outcomes in patient care. Since tumor response criteria for volumetric measurements are not yet validated, combining the clinical outcome in a larger prospective study group could provide standardized volumetric measurements.

## Supporting Information

S1 Fig Measurement according to RECIST in one dimension. Long axis and short axis measurement in cm is shown in the figure.
(DOCX)

S2 Fig Vitrea® semi-automatic volumetric analysis. Contour of the retroperitoneal lesion is marked.
(DOCX)

S3 Fig Distribution of retroperitoneal lymph nodes. RPAO: right para-aortic, LPAO: left para-aortic, CLRV: caudal left renal vein, CAV: vena cava, AOB: aortic bifurcation.
(DOCX)

S4 Fig Bland Altman plots. **(A)** Display Bland Altman plot observer 1 and 2 volumetric method (cc). (B) Display Bland Altman plot observer 1 and R (radiologist) volumetric method (cc). (C) Display Bland Altman plot observer 2 and R volumetric method (cc). (D) Display Bland Altman plot observer 1 and 2 manual method (mm). (E) Display Bland Altman plot observer 1 and R manual method (mm). (F) Display Bland Altman plot observer 2 and R manual method (mm). Mean difference and limits of agreement are displayed as reference lines.
(DOCX)

S1 Table Patient characteristics and lesion distribution. RPAO: right para-aortic, CLRV: caudal left renal vein, LPAO: left para-aortic, CAV: vena cava, AOB: aortic bifurcation.
(DOCX)

S2 Table Distribution of manual and volumetric lymph node measurements (mm). (DOCX)

S3 Table Overview intra class correlation values between observers across methods. (DOCX)
