# Supplementary material for: Assessment of Volumetric versus Manual Measurement in Disseminated Testicular Cancer; No Difference in Assessment between Non-Radiologists and Genitourinary Radiologist
Source: PLoS One. 2017 Jan 12;12(1):e0168977. doi: 10.1371/journal.pone.0168977 (PMC5230761; doi:10.1371/journal.pone.0168977)
Supplement: S2 Fig — Contour of the retroperitoneal lesion is marked. (DOCX) [file pone.0168977.s002.docx]

**S2 Fig. Vitrea® semi-automatic volumetric analysis.**

Contour of the retroperitoneal lesion is marked.
