# Supplementary material for: Assessment of Volumetric versus Manual Measurement in Disseminated Testicular Cancer; No Difference in Assessment between Non-Radiologists and Genitourinary Radiologist
Source: PLoS One. 2017 Jan 12;12(1):e0168977. doi: 10.1371/journal.pone.0168977 (PMC5230761; doi:10.1371/journal.pone.0168977)
Supplement: S3 Fig — RPAO: right para-aortic, LPAO: left para-aortic, CLRV: caudal left renal vein, CAV: vena cava, AOB: aortic bifurcation. (DOCX) [file pone.0168977.s003.docx]

**S3 Fig. Distribution of retroperitoneal lymph nodes.**

RPAO: right para-aortic, LPAO: left para-aortic, CLRV: caudal left renal vein, CAV: vena cava, AOB: aortic bifurcation.
