# Supplementary material for: Assessment of Volumetric versus Manual Measurement in Disseminated Testicular Cancer; No Difference in Assessment between Non-Radiologists and Genitourinary Radiologist
Source: PLoS One. 2017 Jan 12;12(1):e0168977. doi: 10.1371/journal.pone.0168977 (PMC5230761; doi:10.1371/journal.pone.0168977)
Supplement: S4 Fig — (A) Display Bland Altman plot observer 1 and 2 volumetric method (cc). (B) Display Bland Altman plot observer 1 and R (radiologist) volumetric method (cc). (C) Display Bland Altman plot observer 2 and R volumetric method (cc). (D) Display Bland Altman plot observer 1 and 2 manual method (mm). (E) Display Bland Altman plot observer 1 and R manual method (mm). (F) Display Bland Altman plot observer 2 and R manual method (mm). Mean difference and limits of agreement are displayed as reference lines. (DOCX) [file pone.0168977.s004.docx]

**S4 Fig. Bland Altman plots. (A)** Display Bland Altman plot observer 1 and 2 volumetric method (cc). (B) Display Bland Altman plot observer 1 and R (radiologist) volumetric method (cc). (C) Display Bland Altman plot observer 2 and R volumetric method (cc). (D) Display Bland Altman plot observer 1 and 2 manual method (mm). (E) Display Bland Altman plot observer 1 and R manual method (mm). (F) Display Bland Altman plot observer 2 and R manual method (mm). Mean difference and limits of agreement are displayed as reference lines.
