# Supplementary material for: Assessment of Volumetric versus Manual Measurement in Disseminated Testicular Cancer; No Difference in Assessment between Non-Radiologists and Genitourinary Radiologist
Source: PLoS One. 2017 Jan 12;12(1):e0168977. doi: 10.1371/journal.pone.0168977 (PMC5230761; doi:10.1371/journal.pone.0168977)
Supplement: S1 Table — RPAO: right para-aortic, CLRV: caudal left renal vein, LPAO: left para-aortic, CAV: vena cava, AOB: aortic bifurcation. (DOCX) [file pone.0168977.s005.docx]

**S1 Table. Patient characteristics and lesion distribution.**

RPAO: right para-aortic, CLRV: caudal left renal vein, LPAO: left para-aortic, CAV: vena cava, AOB: aortic bifurcation.
